# Rheological Investigation as Tool to Assess Physicochemical Stability of a Hyaluronic Acid Dermal Filler Cross-Linked with Polyethylene Glycol Diglycidyl Ether and Containing Calcium Hydroxyapatite, Glycine and L-Proline

**DOI:** 10.3390/gels8050264

**Published:** 2022-04-23

**Authors:** Nicola Zerbinati, Maria Chiara Capillo, Sabrina Sommatis, Cristina Maccario, Giuseppe Alonci, Raffaele Rauso, Hassan Galadari, Stefania Guida, Roberto Mocchi

**Affiliations:** 1 Department of Medicine and Surgery, University of Insubria, 21100 Varese, Italy; nicola.zerbinati@uninsubria.it; 2 UB-CARE S.r.l. Spin-Off University of Pavia, 27100 Pavia, Italy; mariachiara.capillo@ub-careitaly.it (M.C.C.); sabrina.sommatis@ub-careitaly.it (S.S.); cristina.maccario@ub-careitaly.it (C.M.); 3 Matex Lab S.p.A, 1228 Geneve, Switzerland; giuseppe.alonci@neauvia.com; 4 Maxillofacial Surgery Unit, Department of Medicine and Surgery, University of Campania “Luigi Vanvitelli”, 80138 Naples, Italy; raffaele.rauso@unicampania.it; 5 College of Medicine and Health Sciences, United Arab Emirates University, Al Ain P.O. Box 15551, United Arab Emirates; hgaladari@uaeu.ac.ae; 6 Dermatology Unit, Department of Surgical, Medical, Dental and Morphological Sciences Related to Transplant, Oncology and Regenerative Medicine, University of Modena and Reggio Emilia, 41124 Modena, Italy; stefania.guida@unimore.it

**Keywords:** rheology, dermal filler, aesthetic medicine, stability, thermal study, viscosity, hyaluronic acid, CaHA, physicochemical characterization, hydrogel

## Abstract

(1) Background: Dermal fillers are commonly used in aesthetic practice and their rheological characterization is of much interest today, as well as the stability study of the finished formula against external stimuli of a different nature (biological and physicochemical). Rheological tools have been exploited to characterize the physiochemical behaviour of a hyaluronic acid (HA) based dermal filler subjected to different thermal conditions over time. The collected results provide an index of its rheological stability. (2) Methods: After a preliminary Amplitude sweep test, the Frequency sweep test was performed in order to study the stability of a HA dermal filler cross-linked with Polyethylene Glycol Diglycidyl Ether (PEGDE) and containing Calcium Hydroxyapatite (CaHA), Glycine and L-Proline subjected to different conditions. Also, a shear rate ramp test was performed in order to investigate the filler’s flow behavior. (3) Results and Conclusions: G’ (elastic modulus), G’’ (viscous modulus) and consequentially tan δ (tangent of the phase angle) show a similar trend at different thermal conditions, underlining that the product is not affected by the storage conditions. The viscosity of the dermal filler decreases with an increasing shear rate, so a non-Newtonian shear thinning pseudoplastic behavior was demonstrated in all tested conditions.

## 1. Introduction

Aesthetic medicine is a dynamic world in constant development, with the greatest challenge being obtaining new innovative products that allow for ever more accurate and precise applications. Hydrogels are a polymeric semisolid material with a three-dimensional (3D) architecture, with mesh structure and highly interconnected porosity [1]. They show high water content and good biocompatibility, and for their physical similarities to human tissues, hydrogels have been commonly used for aesthetic purposes, as cell culture substrates and wound dressings, for drug delivery, biosensing and bioimaging in response to the complex environment of the human body, so as to carry out the accurate diagnosis and treatment of a variety of diseases [2]. The study of a new formulation requires multiple steps to demonstrate safety, efficacy and stability in order to guarantee the protection and satisfaction of the consumer. During formulation, filling, usage and storage, products are exposed to possible external factors such as physical, microbiological and chemical influences which can lead to instability with different grades. Microbiological instabilities are caused by fungi, yeast or bacteria contaminations, while physical and chemical changes in the formulation are influenced, for example, by temperature, light and interactions of the ingredients with the packaging or with each other [3]. Since thermodynamic influences may affect the storage stability, accelerating storage conditions with temperature variation in order to induce rapid physical and chemical alterations in the product represents a valid way to obtain stability performance prediction [4]. The rheological characterization of the hyaluronic acid (HA) dermal filler allows us to study the physical stability, aesthetical outcome, quality and usefulness. A thermal stress can alter the product’s viscosity and storage and loss modulus, so rheological studies can grant useful insight into the material behavior under different temperature stresses [5]. In particular, rheology allows for understanding and evaluating the response of the material to different stimuli, such as stress or deformation at various frequencies, and at different temperatures and conditions. Rheology has also become a tool to support safer developments of the product and to quickly reproduce the consumer sensation during the administration or the application of the hydrogel. The rheological characterization is also useful for evaluating the consistency of the product, as well as the effect of the stabilizers on the formulation itself [6]. Hydrogels have rheological properties that allow them to be classified as viscoelastic, as they exhibit both elastic and viscous behavior and present physical properties between liquid and solid states. Through the frequency sweep test, it is possible to observe how the relationship between the viscous and elastic moduli evolves in function of the frequency of the applied stress. The test is performed within the Linear Viscoelastic Region (LVER), determined by a preliminary Amplitude sweep test at a constant frequency, in order to avoid sample damage. In this range, G’ (elastic modulus), G’’ (viscous modulus) and tan δ (tangent of the phase angle) do not vary with the application of deformation [7].

The viscosity of a dermal filler is related to the concentration of not-crosslinked and crosslinked HA, to the degree of crosslinking, to the molecular weight distribution, to the average gel particle size and to the manufacturing process [8]. HA dermal fillers need to be formulated in order to have gel particles that, at low frequencies, retain elasticity (stiffness). It is also crucial that HA hydrogels have low viscosity at high shear (100 s^−1^), so they can be extruded through a small-gauge needle. High viscosity under low-shear rates is indeed comparable to the condition of the hydrogel after injection or when at rest in the packaging (shear rate 0.1 s^−1^) [9]. In our study, we use rheology as tool for the stability assessment of a HA-based dermal filler cross-linked with polyethylene glycol diglycidyl ether (PEGDE), containing 26 mg/mL HA, 0.1% of Glycine and L-Proline and 1% Calcium Hydroxiapatite (CaHA) (Matex Lab S.p.a. Brindisi, Italy). Amplitude and Frequency sweep tests were performed to verify that the rheological parameters maintain a similar trend when the samples are subjected to different thermal stimuli over time. The evaluation of the viscoelasticity trend can be used as an index of the formulation stability when subjected to external stimuli. Characterization through shear rate is instead used as a tool to investigate the ability of the hydrogel to flow with a non-Newtonian shear-thinning behavior. PEGDE is a cross-linker agent widely used to modify and improve the physical properties of the linear chain of HA, conferring a 3D more stable structure [10]. The cross-linking reaction consists of an epoxide ring opening with the hydroxyl group of the HA in an alkaline environment that leads to the formation of a stable ether bond while, at the same time, maintaining the biocompatibility and biological activity of the native HA [11]. The filler used in the present study also contains Glycine and L-Proline, two amino acid constituents of all the types of collagen [12]. The use of CaHA microspheres for aesthetic purposes is closely related to its ability to provide a non-permanent volumizing effect for revolumization and tissue support. CaHA plays a key role in the rheological profile of the HA-based dermal fillers and provides a higher viscosity (η) and elastic modulus (G’) than HA fillers, conferring a scientific basis for the observed ability of CaHA in tissue support and facial revolumization [13]. This filler has an important clinical indication and high level of biosafety, as shown in a precedent laboratory evaluation [14].

## 2. Results and Discussion

### 2.1. Frequency Sweep Test

**Figure 1:**
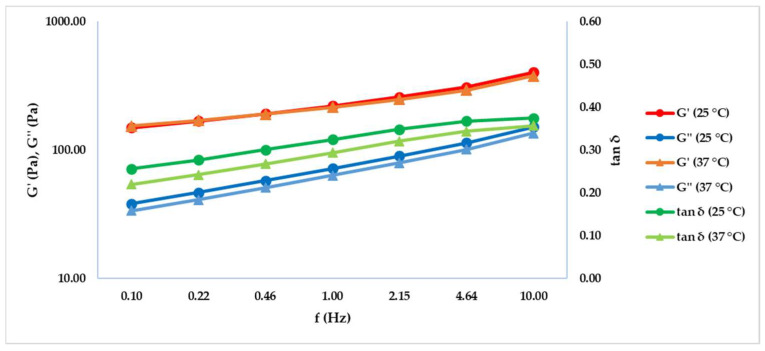
G’, G’’ and tan δ trends obtained by the frequency sweep test at 25 and 37 °C in the range between 0.1 and 10 Hz.

Frequency sweep test results obtained at 25 and 37 °C are shown in Table 1, Table 2 and summarized graphically in Figure 1. The test was performed in order to mimic the dermal fillers’ behavior when stored at different temperatures or when injected into the living tissue. Figure 1 shows that G’ and G’’ increase proportionally with the frequency value. G’ is always greater than G’’ both at 25 and 37 °C, and the obtained tan δ value is always less than unity, confirming a solid-like behavior of the hydrogels at all the frequencies and high degree of crosslinking. G’ and G’’ run parallel without a crossover point, so the crosslinked gel’s elastic nature prevailed on the viscous nature under the conditions tested.

### 2.2. Rheology as Tool for the Study of Dermal Filler Behaviour at Different Temperatures

**Figure 2:**
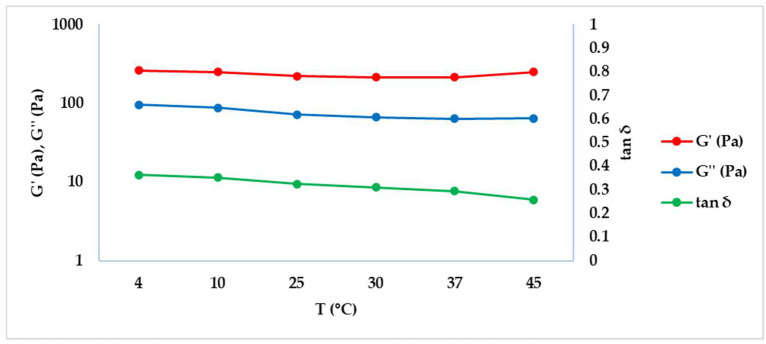
G’, G’’ and tan δ trends obtained by the frequency sweep test at different temperatures.

The frequency sweep test was performed at different temperatures (4, 10, 25, 30, 37, 45 °C), without unloading the samples, at shear strain 1%, obtained by the preliminary Amplitude sweep test [11]. Figure 2 describes the similar trend of G’ (elastic modulus) and G’’ (viscous modulus) with increasing temperature while tan δ (tangent of the phase angle) decreases slightly with temperature. Data for each studied temperature were extrapolated at 1 Hz and are summarized in Table 3.

Figure 2 shows that G’ prevails on G’’, underlining the prevalence of the elastic behavior in the crosslinked HA hydrogel. As expected, the tan δ trends summarize the elastic modulus and the viscous modulus course, since it is obtained by the ratio of G’’/G’.

**Figure 3:**
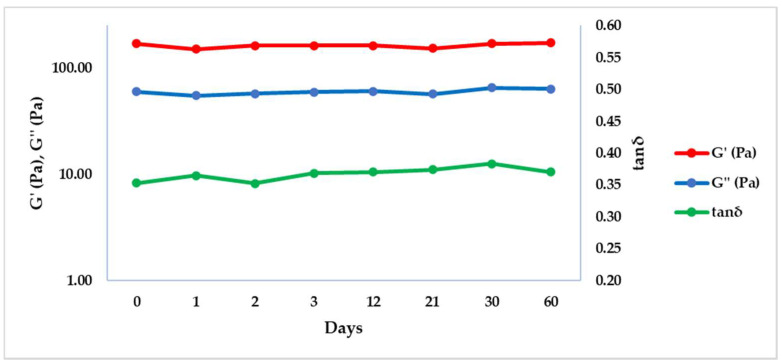
G’, G’’ and tan δ trends obtained by the frequency sweep test at 25 °C and 1 Hz after the conservation of the product in the oven at 50 °C.

**Figure 4:**
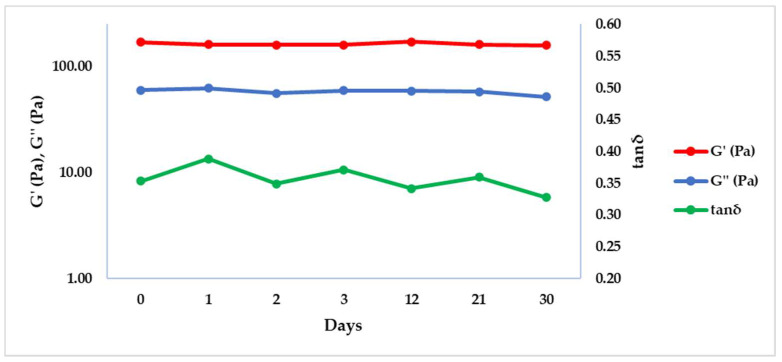
G’, G’’ and tan δ trends obtained by the frequency sweep test at 25 °C and 1 Hz after the conservation of the product in the freezer at −20 °C.

The dermal fillers stability was also evaluated with a frequency sweep test after storing the samples at –20 and 50 °C at different time points (0, 1, 2, 3, 12, 21, 30 and 60 days), in order to simulate the stability of the filler during the storage in a freezer or at high temperature. Results were reported graphically in Figure 3 and Figure 4, while G’, G’’ and tan δ values were extrapolated at 1 Hz and summarized in Table 4 and Table 5, respectively at 50 and −20 °C.

Both at −20 and 50 °C, G’, G’’ and consequentially tan δ remained constant respect to time zero (T0), underlining that the product is not affected by the different thermal conditions of conservation in two months.

The results of this rheological analysis indicate that the thermal stress applied to HA dermal fillers did not affect G’, G’’ or tan δ, and is a good indication of the stability of crosslinked PEGDE hydrogels even in extreme conditions of temperature for prolonged periods.

### 2.3. Dermal Fillers’ Viscosity Determination

**Figure 5:**
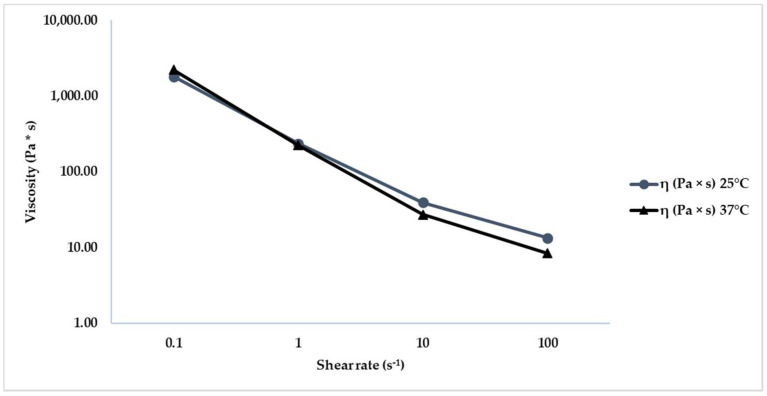
Shear rate ramp test complete trend obtained at 25 °C and 37 °C.

The HA-based hydrogel’s non-Newtonian shear thinning behavior is demonstrated by the viscosity decreases undershear rates. Shear thinning is the most common type of non-Newtonian behavior of fluids, considered synonymous for pseudoplastic behavior and seen in many industrial and everyday applications. The pseudoplastic behavior of dermal fillers is an index of the hydrogel ability to flow when extruded through a small-gauge needle or during implantation when gels are subjected to shear stress and vertical compression/elongation forces. Table 6 shows the higher viscosity at a low shear rate while, increasing the shear rate, the filler becomes less viscous, and the viscosity decreased. Figure 5 shows the HA dermal fillers viscosity’s complete trend, increasing the shear rates both at 25 °C and 37 °C.

## 3. Conclusions

Our overview presents a physiochemical characterization in different stress conditions of a hyaluronic acid dermal filler cross-linked with polyethylene glycol diglycidyl ether and containing calcium hydroxyapatite, glycine and proline. The prevalent elastic profile respects that a viscous nature has been demonstrated by data obtained both at 25 °C (storage/extrusion condition) and 37 °C (injection condition), showing a consistent trend of the cross-linked gel in both case studies. At the same thermal stress conditions, non-Newtonian shear thinning behavior oh the HA hydrogel has been demonstrated by the share rate ramp test. Hyaluronic acid physical properties make its implantation durable and easy because at high shear rates it shows a low viscosity, able to facilitate its injection when forced through a needle, while its high viscosity at low shear rates allows for duration in the tissues. Data obtained show that at low shear rate (simulating storage condition) the dermal filler presents a higher viscosity while, increasing the shear rate (extrusion/application conditions) viscosity decreased. This behavior has been confirmed bot at 25 than at 37 °C. Therefore, the results obtained represent overall, a complete characterization of the dermal filler during its shelf-life, guaranteeing conformity of its rheological profile from packaging to final use. Physicochemical stability of the HA hydrogel has also been confirmed by data obtained in the most extreme thermal conditions (from−20 to 50 °C) for a total period of 60 days, demonstrating a satisfactory stability of the formulation under the tested stress conditions, but also over time.

## 4. Materials and Methods

### 4.1. Frequency Sweep Test

A preliminary Amplitude sweep test (supplementary materials, Figure S1 and Table S1) was performed in order to define a shear strain value to perform a frequency sweep test that allows us to determine G’ (elastic modulus), G’’ (viscous modulus) and tan δ (the angle phase’s tangent). The test was performed at a working gap of 1.0 mm, using a 20 mm plate-plate geometry (Malvern Panalytical, Worcestershire, UK, PU20 SR2467 SS) in the frequency range from 0.1 to 10 Hz. In order to remain in the LVER (determined with the preliminary amplitude sweep test) and limit the gel’s system damage, the shear strain was set up at 1% [11]. In order to predict the behavior at rest or in tissues, results obtained at 25 and 37 °C were extrapolated at 1 Hz based on Lorenc et al. consideration that it is in the range of parameters relevant for facial and physiological skin movements [15,16,17].

Each run was performed in triplicate. Rheological analyses were performed using Kinexus Plus Rheometer (Malvern Panalytical, Worcestershire, UK) while data processing was performed using rSpace for Kinexus software version 1.76 (Malvern Panalytical).

### 4.2. Dermal Fillers’ Behaviour at Different Temperatures

To evaluate the dermal filler rheological properties at different temperatures, a frequency sweep was performed at 4, 10, 25, 30, 37 and 45 °C, setting up an internal method [16]. The test was performed with a 20 mm plate-plate geometry and a working gap of 1.0 mm in the frequency range of 0.1 to 10 Hz and at 1% shear strain. Results were extrapolated at 1 Hz. To avoid loading errors, the same sample was used for the whole temperature range, taking care to allow for an equilibration time at each temperature for 5 min before starting the measure. The test was performed in triplicate.

To evaluate the hydrogel stability, the dermal fillers were stored in their original packaging at −20 and 50 °C and analyzed at different time points (0, 1, 2, 3, 12, 21, 30 and 60 days). The sample was removed from the freezer or from the oven at the appropriate time point, and a frequency sweep test was performed as described above, at a temperature of 25 °C. The test was performed in duplicate.

### 4.3. Dermal Fillers’ Viscosity Determination

In order to investigate the Hyaluronic acid dermal filler’s flow behavior, a sequence shear rate ramp was performed at a fixed temperature (25 °C and 37 °C) with a rotational rheometer Kinexus Plus (Malvern Panalytical, Worcestershire, UK). The test was performed with 7 points for decade, 1 mm of working gap, 20 mm plate-plate geometry (PU20 SR2467 SS) and into a wide range of shear rates values (0.1–100 s^−1^ logarithmic scale) to simulate the behavior of the gel at rest and during extrusion. The Shear rate ramp test measures shear viscosity (η) with increasing shear rates in order to determine if a material is Newtonian or non-Newtonian [18,19]. The test was performed in triplicate, and data processing was performed using rSpace for Kinexus software version 1.76 (Malvern Panalytical).

## Figures and Tables

**Table 1:** Results of G’, G’’ and tan δ were obtained by the frequency sweep test at 25 °C. Results are reported as average ± standard deviation (SD).

T (°C)	f (Hz)	G’ (Pa) ± SD	G’’ (Pa) ± SD	tan δ ± SD
25	0.10	149.40 ± 8.61	38.30 ± 4.03	0.26 ± 0.02
25	0.22	168.33 ± 10.13	46.71 ± 4.62	0.28 ± 0.02
25	0.46	191.70 ± 12.16	57.71 ± 5.51	0.30 ± 0.02
25	1.00	220.90 ± 14.62	71.85 ± 6.56	0.33 ± 0.01
25	2.15	258.30 ± 17.71	90.09 ± 7.87	0.35 ± 0.01
25	4.64	309.83 ± 21.47	113.90 ± 9.45	0.37 ± 0.01
25	10.00	403.40 ± 24.50	151.1 ± 13.75	0.37 ± 0.02

**Table 2:** Results of G’, G’’ and tan δ obtained by the frequency sweep test at 37 °C. Results are reported as average ± standard deviation (SD).

T (°C)	f (Hz)	G’ (Pa) ± SD	G’’ (Pa) ± SD	tan δ ± SD
37	0.10	153.70 ± 8.00	33.78 ± 2.23	0.22 ± 0.02
37	0.22	170.03 ± 8.17	41.13 ± 2.75	0.24 ± 0.02
37	0.46	190.23 ± 8.57	50.90 ± 3.25	0.27 ± 0.02
37	1.00	215.57 ± 9.35	63.42 ± 3.95	0.29 ± 0.02
37	2.15	248.17 ± 10.57	79.64 ± 4.89	0.32 ± 0.01
37	4.64	293.67 ± 12.30	101.07 ± 5.95	0.34 ± 0.01
37	10.00	378.73 ± 13.48	135.23 ± 8.57	0.36 ± 0.01

**Table 3:** G’, G’’ and tan δ values obtained at 1 Hz by the Frequency sweep test at different temperatures. Results are reported as average ± standard deviation (SD).

T (°C)	G’ (Pa) ± SD	G’’ (Pa) ± SD	tan δ ± SD
4	261.83 ± 17.96	95.43 ± 8.35	0.36 ± 0.01
10	249.70 ± 18.38	88.08 ± 7.95	0.35 ± 0.01
25	220.90 ± 14.62	71.85 ± 6.56	0.32 ± 0.01
30	214.97 ± 12.16	66.89 ± 5.66	0.31 ± 0.01
37	215.57 ± 9.35	63.42 ± 3.95	0.29 ± 0.02
45	251.03 ± 9.46	64.67 ± 2.77	0.26 ± 0.02

**Table 4:** Results of G’, G’’ and tan δ obtained by the frequency sweep test at 1 Hz at 25 °C after the conservation of the product in the oven at 50 °C. Results are reported as average ± standard deviation (SD).

T (°C)	Time (Day)	G’ (Pa) ± SD	G’’ (Pa) ± SD	tan δ ± SD
50	0	169.55 ± 0.21	59.90 ± 0.72	0.35 ± 0.00
50	1	150.40 ± 5.94	54.98 ± 6.14	0.37 ± 0.03
50	2	162.55 ± 14.35	57.46 ± 8.87	0.35 ± 0.02
50	3	161.95 ± 22.56	59.49 ± 5.06	0.37 ± 0.02
50	12	162.25 ± 8.56	60.20 ± 7.91	0.37 ± 0.03
50	21	152.90 ± 21.07	56.92 ± 4.21	0.37 ± 0.02
50	30	170.00 ± 27.15	65.16 ± 10.41	0.38 ± 0.00
50	60	171.50 ± 11.03	63.39 ± 1.03	0.37 ± 0.02

**Table 5:** Results of G’, G’’ and tan δ obtained by the frequency sweep test at 1 Hz at 25 °C after storage of the product at –20 °C. Results are reported as average ± standard deviation (SD).

T (°C)	Time (Day)	G’ (Pa) ± SD	G’’ (Pa) ± SD	tan δ ± SD
−20	0	169.55 ± 0.21	59.90 ± 0.72	0.35 ± 0.00
−20	1	160.90 ± 15.70	62.45 ± 4.89	0.39 ± 0.01
−20	2	159.47 ± 3.61	55.65 ± 4.17	0.35 ± 0.03
−20	3	160.50 ± 11.60	59.52 ± 2.57	0.37 ± 0.01
−20	12	171.75 ± 13.93	58.58 ± 2.61	0.34 ± 0.01
−20	21	160.80 ± 1.70	57.79 ± 0.21	0.36 ± 0.00
−20	30	150.40 ± 8.49	50.11 ± 1.18	0.33 ± 0.03
−20	60	169.00 ± 3.39	60.41 ± 0.18	0.36 ± 0.01

**Table 6:** Shear rate ramp test values obtained in order to simulate different conditions at 25 °C and 37 °C. Results are reported as average value ± standard deviation (SD).

Condition of the Product	Temperature (°C)	Shear Rate (s^−1^)	η (Pa × s) ± SD
Rest	25	10^−2^	1811.33 ± 25.74
Extrusion	25	10^2^	13.43 ± 1.81
Application	37	10^2^	8.39 ± 0.30

## Data Availability

Data are included in the text; raw data are available from the corresponding author.

## Supplementary Materials

The following supporting information can be downloaded at: https://www.mdpi.com/article/10.3390/gels8050264/s1, Figure S1: Rheological characterization of the 26 mg/mL HA hydrogel containing CaHA, Glycine and L-Proline (Matex Lab S.p.A.) obtained at 25 and 37 °C in the linear viscoelastic region (LVER). Data are represented as average ± SD, Table S1: Rheological characterization of the 26 mg/mL HA hydrogel containing CaHA, Glycine and L-Proline (Matex Lab S.p.A.) obtained at a fixed shear strain (1%) and temperature (25 and 37 °C) in the linear viscoelastic region (LVER). Data are represented as average ± standard deviation (SD).
