# Supplementary material for: Rheological Investigation as Tool to Assess Physicochemical Stability of a Hyaluronic Acid Dermal Filler Cross-Linked with Polyethylene Glycol Diglycidyl Ether and Containing Calcium Hydroxyapatite, Glycine and L-Proline
Source: Gels. 2022 Apr 23;8(5):264. doi: 10.3390/gels8050264 (PMC9140203; doi:10.3390/gels8050264)
Supplement: Supplementary file 1 [file gels-08-00264-s001.zip › gels-1661656-supplementary.pdf]

gels

Article

Rheological Investigation as Tool to Assess Physicochemical Stability of a Hyaluronic Acid Dermal Filler Cross-Linked with Polyethylene Glycol Diglycidyl Ether and Containing Calcium Hydroxyapatite, Glycine and L-Proline

Nicola Zerbinati, Maria Chiara Capillo, Sabrina Sommatis, Cristina Maccario, Giuseppe Alonci, Raffaele Rauso, Hassan Galadari, Stefania Guida and Roberto Mocchi

Supplementary Materials

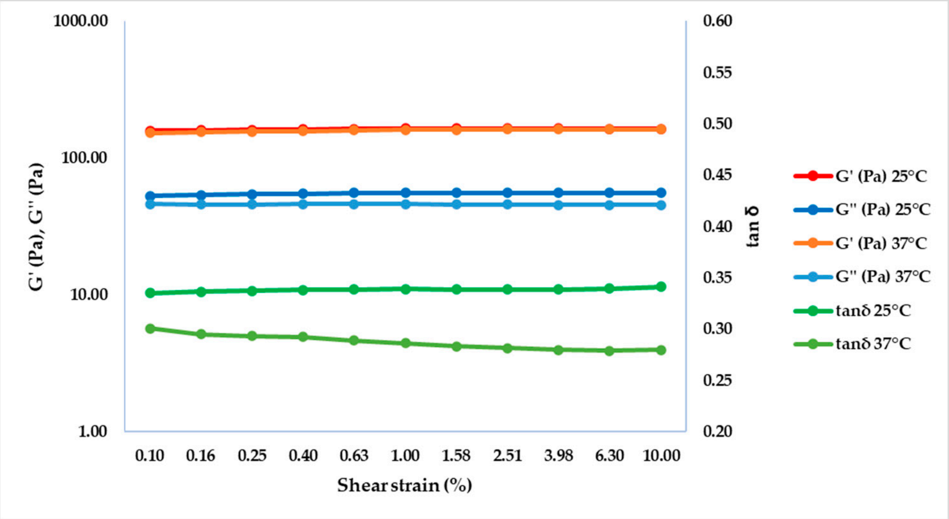

Figure S1. Rheological characterization of the 26 mg/mL HA hydrogel containing CaHA, Glycine and L-Proline (Stimulate, Matex Lab S.p.A.) obtained at 25 and 37 °C in the linear viscoelastic region (LVER). Data are represented as average ± SD.

Table S1. Rheological characterization of the 26 mg/mL HA hydrogel containing CaHA, Glycine and L-Proline (Stimulate, Matex Lab S.p.A.) obtained at a fixed shear strain (1%) and temperature (25 and 37 °C) in the linear viscoelastic region (LVER). Data are represented as average ± standard deviation (SD).

Temperature	G′ (Pa)	G′′ (Pa)	tanδ
25 °C	164.67 ± 2.94	55.84 ± 5.07	0.34 ± 0.03
37 °C	161.17 ± 4.68	46.23 ± 6.87	0.29 ± 0.03
